# Sesamin attenuates intestinal injury in sepsis via the HMGB1/TLR4/IL-33 signalling pathway

**DOI:** 10.1080/13880209.2020.1787469

**Published:** 2020-09-06

**Authors:** Zhi-Ling Li, Min Gao, Ming-Shi Yang, Xue-Fei Xiao, Jing-Jing Liu, Bing-Chang Yang

**Affiliations:** a Translational Medicine Center of Sepsis, The Third Xiangya Hospital of Central South University, Changsha, PR China; b Department of Critical Care Medicine, The Third Xiangya Hospital of Central South University, Changsha, PR China

**Keywords:** Intestine, CLP, inflammation, MPO, MDA, DAO

## Abstract

**Context:**

Sepsis is currently one of the leading causes of death in intensive care units (ICUs). Sesamin was previously reported to inhibit inflammation. However, no studies have revealed the impact of sesamin on sepsis.

**Objective:**

We studied the mechanism underlying the effect of sesamin on the pathophysiology of sepsis through the HMGB1/TLR4/IL-33 signalling pathway.

**Materials and methods:**

Fifty male BALB/c mice (*n* = 10 per group) were used to establish a caecal ligation and puncture (CLP) mouse model, and given daily injections of sesamin at a low, middle, or high concentration (25, 50, or 100 μM) during the seven-day study period; survival curves were generated by the Kaplan–Meier method. H&E staining and TUNEL staining were performed to assess changes in intestinal morphology intestinal damage in the mouse intestinal epithelium. Molecules related to the HMGB1/TLR4/IL-33 pathway were assessed by RT-qPCR and Western blotting.

**Results:**

We found mice in the sepsis group survived for only 4 days, while those treated with sesamin survived for 6–7 days. In addition, sesamin significantly relieved the increase in the levels of MPO (21%, 33.3%), MDA (40.5% and 31.6%), DAO (1.24-fold and 2.31-fold), and pro-inflammatory cytokines such as TNF-α (75% and 79%) and IL-6 (1-fold and 1.67-fold) 24 and 48 h after sepsis induction and downregulated the expression of HMGB1, TLR4, and IL-33 while upregulating the expression of ZO-1 and occludin.

**Discussion and conclusions:**

Sesamin improved the 7-day survival rate of septic mice, suppressed the inflammatory response in sepsis through the HMGB-1/TLR4/IL-33 signalling pathway, and further alleviated intestinal injury.

## Introduction

Sesamin is a lignin component isolated from sesame oil and is commonly considered a healthy food with antihypertensive, anti-inflammatory, antiviral and antioxidative properties (Xu et al. 2015). Additionally, the potential anticancer properties of sesamin in lung cancer, cervical cancer, and colon cancer have been reported in recent years (Dou et al. 2018; Cavuturu et al. 2019; Fang et al. 2019). Previous evidence has shown that sesamin can inhibit lipopolysaccharide (LPS)-induced expression of Toll-like receptor (TLR) 4, which further results in repression of the inflammatory response (Qiang et al. 2016). Additionally, sesamin has been reported to suppress the production and secretion of pro-inflammatory cytokines such as tumour necrosis factor-α (TNF-α) and interleukin-6 (IL-6) (Ozcan et al. 2004) by mast cells in a manner dependent on their regulatory roles in the p38 MAPK/NF-κB signalling pathway (Li et al. 2016). Currently, sepsis is the leading cause of death among adults in non-coronary intensive care units (ICUs). Data from the Centres for Disease Control (CDC) showed that in 2013, sepsis had become one of the top 10 causes of death with a high mortality rate in America (Milbrandt et al. 2008; Khailova et al. 2013). Sepsis-associated clinical manifestations include systemic inflammatory response syndrome (SIRS), sepsis, severe sepsis and septic shock. SIRS appears to occur more frequently than other manifestations, and causes symptoms including multi-organ injury, disseminated intravascular coagulation, shock, and death (Sursal et al. 2013). The incidence of SIRS ranges from 40% to 70% of all patients admitted to ICUs and is associated with progression to severe complications (Schoenberg et al. 1998).

Translocation of bacteria is the potential initiator of SIRS, and it facilitates the passage of enteric microbes through the permeable intestinal epithelial barrier and ultimately leads to the release of pro-inflammatory cytokines (Strober et al. 2002; Rakoff-Nahoum et al. 2004). TLR4, which is required for this process, plays a vital role in the development of sepsis (Neal et al. 2006). TLR4 was previously shown to be critical for the recognition of the cell component LPS on Gram-positive bacteria (Poltorak et al. 1998; Qureshi et al. 1999). LPS-stimulated TLR4 signalling can activate interleukin-1 receptor (IL-1R)-associated kinase (IRAK) via MyD88 and MD2, which recruits TNF receptor-associated factor 6 (TRAF6) and triggers the activation of the transcription factor NF-κB as well as the release of pro-inflammatory cytokines (Takeda and Akira 2004). Toll-interleukin 1 receptor (TIR) domain-containing adaptor protein (TIRAP) is essential for the MyD88-dependent signalling pathway downstream of TLR2 and TLR4, which stimulates the activation of NF-κB (Yamamoto et al. 2002). It has been reported that TIRAP expression is associated with sepsis-associated acute lung injury (Song et al. 2010). More importantly, evidence has revealed that the protein levels of TLRs and TIRAP are positively related to sepsis severity in septic patients (Zhang et al. 2016), indicating the importance of TLR4 and its relevant signalling during the development of sepsis.

Interleukin-33 (IL-33) (Ozcan et al. 2004) is expressed in the intestinal epithelium, lung and pulmonary endothelium (Moussion et al. 2008). It has been reported that TLR-4 activity is dependent IL-33 signalling in allergic inflammation in mice (Tjota et al. 2013) as well as in acute systemic injury (Chang et al. 2016). As a member of the IL-1 family, IL-33 binds to its corresponding receptor suppression of tumorigenicity 2 (ST2), which is expressed on the surface of mast cells or T helper 2 (Th2) cells, leading to the activation of the p38 MAPK/NF-κB pathway and ultimately enhancing the production of Th2-associated cytokines (Schmitz et al. 2005). It is interesting that IL-33 has been reported to reduce inflammatory cell infiltration in the mouse mucosa and indirectly induce tolerogenic CD103^+^ dendritic cells (DCs). High mobility group box 1 (HMGB1) released by macrophages has been demonstrated to be an important mediator of organ damage in severe sepsis and is expressed at a high level in experimental animal models, causing lethal damage, according to early studies (Wang et al. 1999; Yang et al. 2004). By binding to the TLR4 surface receptor, HMGB1 exerts its effect on inflammation. Evidence has also shown that HMGB1 is involved in TLR4-dependent increases in IL-33 expression (Chang et al. 2017), thus, indicating the potential regulatory role of the HMGB1/TLR4/IL-33 axis in the pathophysiology of sepsis.

However, to date, a few studies have elucidated the effects of sesamin on the progression of sepsis via TLR4-associated signalling. Therefore, in this study, we investigated the roles of sesamin in regulating the development of sepsis via the HMGB1/TLR4/IL-33 signalling pathway by examining its impacts on the production of pro-inflammatory cytokines and the expression of IL-33, HMGB1, TLR4, ZO-1, and occludin.

## Materials and methods

### Reagents and cell treatment

The human colon mucosal epithelial cell line NCM-460 was purchased from American Type Culture Collection (ATCC, Manassas, VA). Sesamin and LPS were purchased from Sigma-Aldrich (St. Louis, MO). The HMGB1 antagonist ammonium glycyrrhizinate (AG, HY-N0184B) was purchased from MedChemExpress (MCE, Monmouth Junction, NJ). Sesamin was initially dissolved in dimethyl sulphoxide (DMSO) and stored at −20 °C and was then thawed to be diluted in the cell culture medium to the required concentrations. NCM-460 cells were maintained in RPMI 1640 (Gibco, Gran Island, NY) with 15% foetal bovine serum (FBS) at 37 °C with 5% CO_2_. To establish an *in vitro* sepsis model, NCM-460 cells were seeded in 96-well plates at a density of 5 × 10^3^ cells/well and cultured overnight. The cells were then incubated alone or with LPS (1 μg/mL), LPS + sesamin (5 μM)/AG (50 µg/mL), or LPS + sesamin + AG as described above for 48 h and then collected for subsequent RT-qPCR and Western blotting analyses.

### Animals

Fifty male BALB/c mice weighing 20 ± 2 g (8–12 weeks of age) were purchased from the Centre of Experimental Animals of Silaike (Shanghai, China). The mice were fed standard fat rodent pellets and quarantined in individual cages under pathogen-free conditions. All animals were treated in accordance with the National Institutes of Health Guidelines for the Care and Use of Laboratory Animals and the Use Committee of Xiangya Hospital, Central South University.

### Construction of the caecal ligation and puncture (CLP) model and experimental setting

Each group was anaesthetized with 4% chloral hydrate, after which a 20 mm middle abdominal incision was made to expose the caecum. In the sepsis group, the caecum of each mouse was punctured twice with a 21-gauge needle to ensure that a small quantity of faeces extruded through the wound. The bowel was then repositioned, the laparotomy incision was closed, and sterile saline solution (0.9%, 24 mL/kg body weight) was administered for fluid resuscitation. In the sham group, mice underwent the same procedure except for ligation and puncture of the caecum. In the sesamin + sepsis group, the mice were given an intraperitoneal injection of sesamin at a low (25 μM), medium (50 μM), or high (100 μM) concentration 30 min before the laparotomy incision was closed. The animals were randomized into five groups: (1) the sham-operated group (*n* = 10); (2) sepsis (CLP/normal saline) group (*n* = 10), in which rats were injected intraperitoneally with normal saline immediately after wound closure; (3) sepsis + sesamin (low) group (*n* = 10), in which rats underwent CLP and were injected with 25 μM sesamin; (4) sepsis + sesamin (medium) group (*n* = 10), in which rats underwent CLP and were injected with 50 μM sesamin; and (5) sepsis + sesamin (high) group (*n* = 10), in which rats underwent CLP and were injected with 100 μM sesamin. Each animal received the same dose of the same agent every day during the 7-day study period. After experimentation, the animals were euthanized by overdose of ketamine and xylazine (>100/10 mg/kg, s.c.) and then subjected to cervical dislocation after 30 min. Intestinal tissues were collected after the mice showed signs of death.

### Histological analysis

To perform morphological analysis of the jejunum mucosa, jejunal tissue specimens were surgically excised, fixed for embedding in paraffin and then stained with haematoxylin and eosin (H&E). Intestinal sections were obtained after routine histological analysis was performed, and photographs were taken under a light microscope (Nikon, Tokyo, Japan; magnifications of 40× and 100×).

### Terminal deoxynucleotidyl transferase dUTP nick end labelling (TUNEL) assay

To assess the apoptosis of colon epithelial cells, a TUNEL assay was performed on tissue specimens from each group. Mouse colon sections were treated with vehicle (DMSO) and fixed with 4% paraformaldehyde (PFA) in PBS for 20 min at room temperature. The sections were then permeabilized with 1% Triton X-100 for 10 min. TUNEL staining was performed with an In Situ Apoptosis Detection Kit (Roche, Basel, Switzerland) according to the manufacturer’s protocol. Sections were treated with DAB for 15 min, and images were obtained with a fluorescence microscope (Leica, Hamburg, Germany). The photographs were observed at a magnification of 100X.

### Measurement of MPO activity

The mice were anaesthetized 24 or 48 h after sepsis induction, and the intestinal tissues were dissected, weighed and homogenized in 0.5% HTAB buffer (hexadecyl trimethyl ammonium bromide in 50 mM potassium phosphate) to obtain a 10% homogenate. The collected intestinal fluid was centrifuged at 5000 rpm for 5 min, after which substrate buffer containing O-dianisidine and 0.0005% hydrogen peroxide was added. MPO activity in intestinal tissue homogenates was determined using a test kit from Nanjing Jiancheng Bioengineering Institute (Nanjing, China). The activity of MPO was measured at a wavelength of 460 nm (Bio-Tek, Winooski, VT).

### Measurement of MDA and DAO in the serum

After mice were subjected to CLP surgery, serum samples were collected from anaesthetized mice, and the supernatant was used to analyze the content of MDA and the activity of DAO. The serum DAO activity and MDA content in the sham, sepsis, and sesamin + sepsis groups were detected at the 24 and 48 h endpoints by using a kit.

### Measurement of cytokine levels in intestinal tissues

Intestinal tissue homogenates (10%) were collected from each group, and the samples were centrifuged at 5000 rpm for 5 min in serum separator tubes and stored at −80 °C for later usage. The concentrations of TNF-α and IL-6 in the sera of septic mice were determined using a multiplex cytokine assay (Bio-Rad, Hercules, CA) according to the manufacturer’s instructions. The plates were read on a microplate reader (Bio-Rad, Hercules, CA) at an absorbance of 450 nm. All tests were performed in triplicate.

### Quantitative real-time PCR (RT-qPCR)

The expression levels of HMGB1, TLR4, IL-33, ZO-1, and occludin were measured using RT-qPCR. Total RNA was isolated from frozen colon tissues using a RNeasy Mini Kit (QIAGEN, Valencia, CA) according to the manufacturer’s protocol. The RNA concentration was confirmed at 260 nm, and purity and integrity were verified using a NanoDrop spectrophotometer. Reverse transcription and cDNA synthesis were performed using 0.2 μg of total RNA. The levels of HMGB1, TLR4, IL-33, ZO-1, and occludin were detected in triplicate using pre-developed TaqMan primers and probes (Applied Biosystems, San Diego, CA) on the ABI Prism7300 Real Time PCR System (Applied Biosystems, San Diego, CA). Relative mRNA abundance was expressed as fold change compared to mRNA levels in the control sham group and was normalised to β-actin. The sequences of PCR primers were as follows:

IL-33 F: 5′-CAATGTTGACGACTCTGGAAAAG-3′,
IL-33 R: 5′-GGGACTCATGTTCACCATCAG-3′;
HMGB1 F: 5′-GGAGTGGCTTTTGTCCCTCAT-3′,
HMGB1 R: 5′-TGCCTCTCGGCTTTTTAGGA-3′;
TLR-4 F: 5′-ACCTGGCTGGTTTACACGTC-3′,
TLR-4 R: 5′-CTGCCATATACATTGCAGAA-3′;
ZO-1 F: 5′-ACCCTCCTTACTCACCACAAG-3′,
ZO-1 R: 5′-ATGAGGCTTCTGCTTTCTGTTG-3′;
occludin F: 5′-AGAGTACATGGCTGCTGCTG-3′,
occludin R: 5′-CACCATCCTCTTGATGTGCG-3′;
β-actin F: 5′-CACCAACTGGGACGACAT-3′,
β-actin R: 5′-ACAGCCTGGATAGCAACG-3′.

### Western blotting

Frozen colon samples and NCM-460 cells from each group were homogenized in a 5× volume of homogenization buffer (Tris HCl, 50 mM; pH, 7.4; NaCl, 100 mM; EDTA, 10 mM; and Triton X-100, 0.5%) with protease inhibitors (Roche, Mannheim, Germany) at 4 °C. The homogenates were centrifuged at 10,000 rpm for 5 min at 4 °C, and the supernatants were collected. Total protein concentration was measured by the Bradford protein assay (Bio-Rad, Hercules, CA). Proteins were lysed with 1× SDS loading buffer, and the lysates were boiled at 100 °C for 5 min and centrifuged at 10,000 rpm for 1 min. Approximately 40 μg of total protein was loaded onto an SDS-PAGE gel and resolved at 120 V for 1–2 h. After that, the proteins were transferred to a PVDF membrane at 300 mA for 2.5 h. The membranes were then washed with 1× TBST 3 times for 10 min each and incubated with secondary antibodies at room temperature for 1 h. Finally, the membranes were incubated with ECL and exposed. The following antibodies from Cell Signaling Technology (USA) were used: anti-HMGB1, anti-TLR4, anti-IL-33, anti-ZO-1 and anti-occludin.

### Statistical analysis

Each experiment was performed three times. All values are presented as the mean ± standard deviation (SD). Survival curves were generated by the Kaplan–Meier method. Differences between two groups were analyzed by Student’s *t*-test, and differences between more than two groups were analyzed by ANOVA followed by a Bonferroni *post hoc* analysis. *p* < 0.05 was considered statistically significant.

## Results

### Sesamin decreases mortality in septic mice

**Figure 1.**
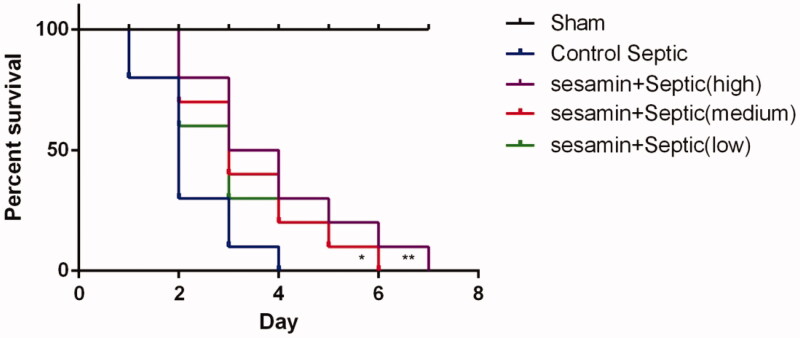
Effect of sesamin on survival in sepsis. (A) Survival curves were generated by the Kaplan–Meier method. The horizontal line represents the sham group. The blue solid line represents the control sepsis group, the green line represents the sesamin (25 µM) + sepsis group, the red line represents the sesamin (50 µM) + sepsis group, and the purple line represents the sesamin (100 µM) + sepsis group. The data are presented as the mean ± SD (*p < 0.05; **p < 0.01, *n* = 10 in each group). Three independent experiments were performed for all tests.

To determine whether sesamin has an effect on mortality in sepsis, mice were subjected to CLP, and the 7-day survival rate was detected. The results showed that the mice in the sepsis group survived for only 4 days and that the mice in the sesamin-treated group (25, 50, and 50 μM) survived for longer than those in the sepsis group. In addition, the survival time of the mice received the medium dose and high doses of sesamin were 28.6% (*p* < 0.05) and 42.9% (*p* < 0.05) longer, respectively, than those of the control mice (Figure 1). This evidence collectively suggests that sesamin can decrease mortality possibly caused by sepsis.

### Sesamin attenuates intestinal injury in sepsis

**Figure 2.**
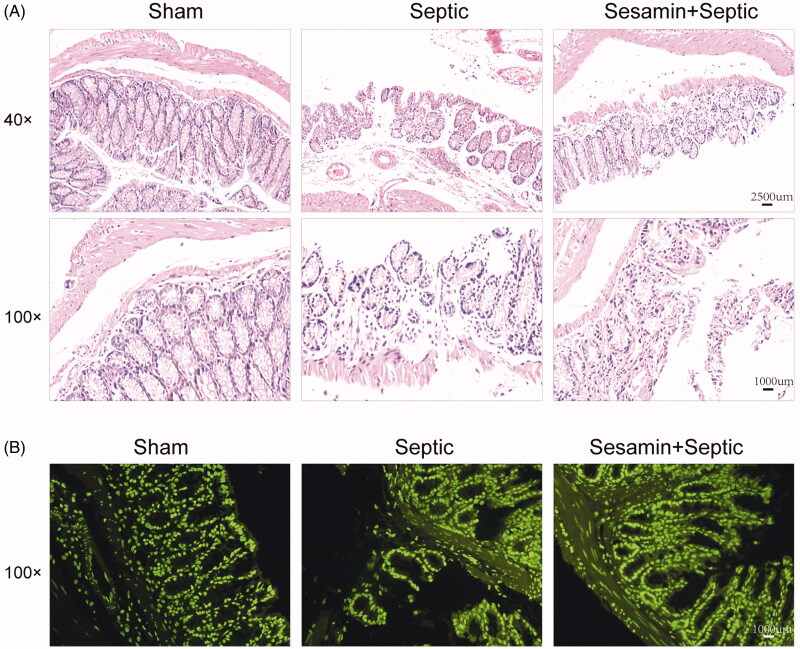
Effect of sesamin on mouse intestinal morphology. (A) H&E staining assay showing intestinal morphology changes (upper: magnification = 40×; bottom: magnification = 100×). (B) TUNEL staining assay showing apoptosis in intestinal tissue specimens (magnification = 100×). Left: sham; middle: control sepsis; right: sesamin + sepsis.

To investigate the effect of sesamin on intestinal injury, pathological examination was performed using H&E staining. As shown in Figure 2(A), mice in the sham group exhibited an integrated intestinal structure and abundant villi epithelial cells as well as crypt cells, and no gap under epithelial cells, haemorrhage necrosis or inflammatory cell infiltration was observed. However, a markedly damaged intestinal structure and enlarged gaps under epithelial cells accompanied by disarranged villi epithelial cells, edoema, increased inflammatory cell infiltration and partial detachment of the epithelial mucosa, which was partly rescued by sesamin, were observed in the sepsis group (Figure 2(A)). Next, we used TUNEL staining to evaluate apoptosis in intestinal tissues among the sham, control sepsis and sesamin + sepsis groups. The results showed a significant increase in the number of apoptotic cells in intestinal tissues from septic mice compared with those from sham mice but a reduction in apoptotic cells in intestinal tissues from mice treated with sesamin compared with those from septic mice (Figure 2(B)), indicating that sesamin can attenuate intestinal injury and decrease apoptosis.

### Sesamin decreases MPO activity and TNF-α and IL-6 levels in the intestine and downregulates DAO activity and MDA content in the serum

**Figure 3.**
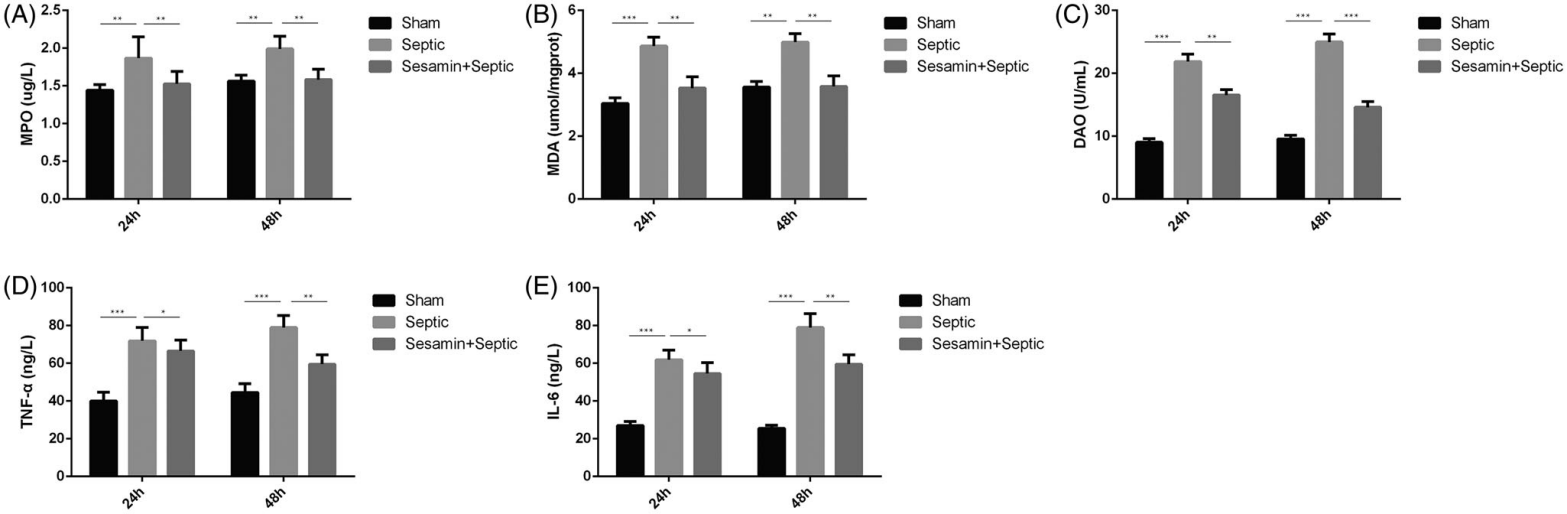
Effect of sesamin on intestinal bacterial clearance and pro-inflammatory cytokine levels in the serum. (A) The level of MPO in intestinal tissue specimens was measured in three groups of mice. (B) The level of MDA in the serum was measured in three groups of mice. (C) The level of DAO in the serum was measured in three groups of mice. (D) The level of TNF-α in the serum was measured in three groups of mice. (E) The level of IL-6 in the serum was measured in three groups of mice. The mean ± SD in the graph presents the relative levels from three independent experiments (*p < 0.05, **p < 0.01, ***p < 0.001, *n* = 3 in each group). Endpoints: 24 h and 48 h. Groups: sham, sepsis, sesamin + sepsis. Sesamin concentration: 50 µM.

The activity of MPO, the levels of pro-inflammatory cytokines, including TNF-α and IL-6, in the intestine, the activity of DAO and the content of MDA in the serum in the sham, sepsis and sesamin (50 μM) + sepsis groups were examined. The activities of DAO (1.24-fold, *p* < 0.001; 2.31-fold, *p* < 0.01), MDA (40.5%, *p* < 0.001; 31.6%, *p* < 0.01) and MPO (21%, *p* < 0.01; 33.3%, *p* < 0.01) were significantly increased in septic mice compared with sham mice at 24 h and 48 h (Figure 3(A–C)). However, sesamin restored the activities of MPA, MDA and DAO at both endpoints. The effects of sesamin on inflammatory cell infiltration in the intestine were measured, and the expression of the pro-inflammatory cytokines TNF-α (75%, 79%, *p* < 0.001) and IL-6 (1-fold, 1.67-fold, *p* < 0.001) was markedly increased in the sepsis group compared with the sham group but significantly decreased in the sesamin-treated group (Figure 3(D,E)) compared with the sham group. In summary, the data suggest that sesamin can lower inflammation.

### Sesamin attenuates the HMGB1/TLR-4/IL-33 signalling pathway in mouse intestinal tissues

**Figure 4.**
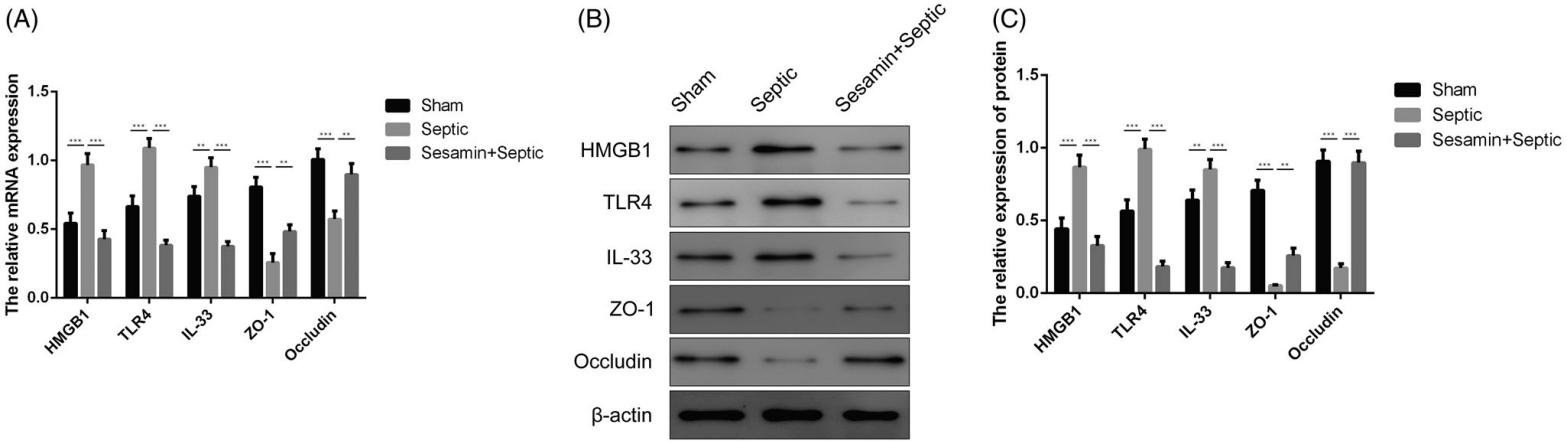
Effect of sesamin on IL-33, HMGB1, TLR4, ZO-1, and occludin expression in colon tissues. (A) RT-qPCR analysis showing the mRNA levels of HMGB1, TLR4, IL-33, ZO-1 and occludin. β-actin was the internal control. The data are presented as the mean ± SD (**p < 0.01; ***p < 0.001). (B) Western blotting analyses showing the protein levels of HMGB1, TLR4, IL-33, ZO-1, and occludin. β-actin was the internal control. (C) Quantitation of HMGB1, TLR4, IL-33, ZO-1, and occludin protein levels according to the western blotting results. The data are presented as the mean ± SD (**p < 0.01; ***p < 0.001). Groups: sham, sepsis, sesamin + sepsis, *n* = 3 in each group. An internal control image was used for illustrative purposes. Three independent experiments were performed for all tests.

To further investigate the molecular mechanism underlying the regulatory roles of sesamin in the inflammatory effects on sepsis, we used RT-qPCR and Western blotting to evaluate the HMGB1/TLR-4/IL-33 signalling pathway. The RT-qPCR results demonstrated that IL-33, HMGB1 and TLR4 were significantly upregulated, while ZO-1 and occludin were downregulated in intestinal tissues from septic mice (Figure 4(A)). However, sesamin markedly downregulated IL-33, HMGB1 and TLR4 and inversely alleviated the reduction in ZO-1 and occludin in septic mice. Similar results were obtained by Western blotting; sesamin significantly reversed the increase in the levels of IL-33, HMGB1 and TLR4 and the decrease in the levels of ZO-1 and occludin observed in the sepsis group (Figure 4(B,C)). Therefore, sesamin pre-treatment effectively attenuated the HMGB1/TLR-4/IL-33 signalling pathway, which was enhanced by sepsis.

### Sesamin suppresses the HMGB1/TLR-4/IL-33 signalling pathway in the NCM-460 cell line

**Figure 5.**
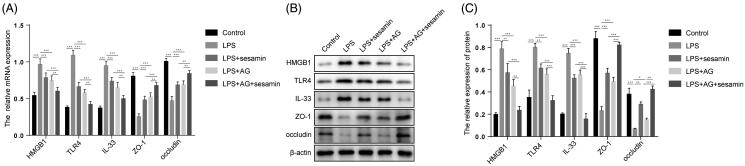
Effect of sesamin on IL-33, HMGB1, TLR4, ZO-1, and occludin expression in the NCM-460 cell line. (A) RT-qPCR analysis showing the mRNA levels of HMGB1, TLR4, IL-33, ZO-1, and occludin in NCM-460 cells. β-actin was the internal control. The data are presented as the mean ± SD (**p < 0.01; ***p < 0.001). (B) Western blotting analyses showing the protein levels of HMGB1, TLR4, IL-33, ZO-1, and occludin in NCM-460 cells. β-actin was the internal control. The data are presented as the mean ± SD (**p < 0.01; ***p < 0.001). Groups: control, LPS, LPS + sesamin, LPS + AG, LPS + AG + sesamin. AG: HMGB1 antagonist. Sesamin concentration: 5 µM. LPS concentration: 1 μg/mL. AG concentration: 50 µg/mL. β-actin was the internal control. Three independent experiments were performed for all tests.

We further investigated whether sesamin can similarly alleviate LPS-induced sepsis *in vitro* through the HMGB1/TLR-4/IL-33 pathway. We used RT-qPCR and Western blotting to examine the expression levels of HMGB1, TLR-4, IL-33, ZO-1, and occludin in NCM-460 cells, and the HMGB1 antagonist AG was used to inhibit the expression of HMGB1. Consistent with the *in vivo* experimental results, LPS intervention remarkably promoted the activation of the HMGB1/TLR-4/IL-33 pathway, as indicated by the increased levels of HMGB1, TLR-4 and IL-33 and decreased levels of ZO-1 and occludin, which were rescued by sesamin pre-treatment, in the LPS-treated group compared to the control group (Figure 5(A,B)). To further substantiate this model, we treated NCM-460 cells with the HMGB1 agonist AG and found that AG treatment effectively antagonized the effect of LPS on the HMGB1/TLR-4/IL-33 pathway (Figure 5(A,B)). This evidence suggests that sesamin can suppress the HMGB1/TLR-4/IL-33 signalling pathway, the activation of which is possibly involved in the *in vitro* LPS-induced sepsis model.

## Discussion

Currently, sepsis is one of the leading causes of death in adults admitted to ICUs, and sesamin, a phytoestrogen abundant in sesame oil, exhibits versatile functions as an anti-inflammatory, antioxidant and anticancer factor (Xu et al. 2015). This study demonstrated that sesamin significantly improved the 7-day survival rate of septic mice in an approximately dose-dependent manner. A higher concentration of sesamin may contribute to better survival in septic mice. Concomitantly, the beneficial effects of sesamin on the survival rate was associated with reduced histological damage to intestinal tissues, alleviation of intestinal apoptosis, and decreased inflammatory cell infiltration and pro-inflammatory cytokine release. Sesamin upregulated the expression of ZO-1 and occludin by downregulating the IL-33/HMGB1/TLR4 signalling pathway.

Based on both morphological and quantitative analyses, this study confirmed that, in addition to having beneficial effects on allergic responses or lung injury, as demonstrated in previous studies, sesamin can reduce sepsis-induced damage to intestinal tissues (Li et al. 2016; Qiang et al. 2016). Since sepsis can cause a systemic inflammatory response and because mounting evidence suggests that the small intestine is critical to the pathophysiology of sepsis (Hassoun et al. 2001; Clark and Coopersmith 2007), the beneficial effects of high-dose sesamin on sepsis-induced intestinal injury in a CLP mouse model indicate its therapeutic role in clinical SIRS.

More importantly, we confirmed that sesamin can decrease the levels of pro-inflammatory cytokines and MPO activity in intestinal tissues as well as DAO and MDA levels in the serum. Inhibition of inflammation was previously reported to alleviate intestinal injury caused by sepsis (Chen et al. 2016). The levels of the pro-inflammatory cytokines IL-6 and TNF-α were downregulated in septic mice, which indicated that sesamin led to a decrease in sepsis-induced inflammation. In addition to having regulatory effects on inflammatory cytokine levels, sesamin was demonstrated to attenuate intestinal oxidative damage by decreasing the levels of DAO, MPO and MDA. MPO and DAO possess bactericidal functions in the mouse small intestine, while their associated product hydrogen peroxide can damage intestinal tissues and enhance inflammation (Hansberry et al. 2017; Koga et al. 2017). In summary, our results suggest that sesamin can act as an anti-inflammatory and antioxidative factor in the intestinal epithelium.

To further elucidate the molecular mechanism underlying the anti-inflammatory functions of sesamin, its potential effects on HMGB1/TLR-4/IL-33 signalling were assessed through RT-qPCR and Western blotting analyses both *in vitro* and *in vivo*. The findings were consistent between the *in vitro* and *in vivo* models; both pre- and post-transcriptional levels of HMGB1, TLR4, and IL-33 were significantly downregulated in the sesamin-treated sepsis group. This confirmed that signalling pathway activation decreased after the administration of sesamin. Our study is the first to show that sesamin can improve sepsis pathophysiology though the TLR4-associated signalling pathway. The interaction between HMGB1 and TLR4 results in the recruitment of TNF-α, which induces the expression of IL-33 and ultimately causes phosphorylation of NF-κB and MAPK activation in mast cells (Schmitz et al. 2005). The inhibitory function of sesamin on this signalling pathway suggests that it can alleviate the severity of sepsis by reducing the inflammatory response in the intestinal epithelium. The restored levels of the tight junction proteins ZO-1 and occludin, which are required for maintaining normal intestinal function, also support this hypothesis.

## Conclusions

This study demonstrated the inhibitory function of sesamin on sepsis-induced intestinal injury. Sesamin increased 7-day survival in septic mice and reduced damage to the structures of epithelial tissues, and the beneficial effects were associated with a higher dose of sesamin. At the molecular level, sesamin may attenuate the HMGB1/TLR4/IL-33 signalling pathway to decrease pro-inflammatory cytokine release both locally and systemically. At the same time, sesamin may restore the levels of ZO-1 and occludin, which are important for the maintenance of normal intestinal function. The present findings provide valuable insights into the potential of sesamin as a new pharmaceutical component to treat sepsis. Moreover, the roles of sesamin in regulating TLR4-associated signalling during the progression of sepsis-induced injury indicate that TLR4 may be a potential pharmaceutical target for the clinical treatment of sepsis in the future.

## Data Availability

All data generated or analyzed during this study are included in this published article.
